# A long-term follow-up study investigating health-related quality of life and resource use in survivors of severe sepsis: comparison of recombinant human activated protein C with standard care

**DOI:** 10.1186/cc6195

**Published:** 2007-12-11

**Authors:** Christopher J Longo, Daren K Heyland, Harold N Fisher, Robert A Fowler, Claudio M Martin, Andrew G Day

**Affiliations:** 1 McMaster University, Main Street West, Hamilton, Ontario, Canada, L8S 4M4; 2 Kingston General Hospital, Queen's University, Stuart Street, Kingston, Ontario, Canada, K7L 2V7; 3 Eli Lilly Canada Inc., Danforth Avenue, Scarborough, Ontario, Canada, M1N 2E8; 4 Sunnybrook Health Sciences Centre, Bayview Avenue, Toronto, Ontario, Canada, M4N 3M5; 5 London Health Sciences Centre, Commissioners Road East, London, Ontario, Canada, N6A 5W9; 6 Clinical Research Centre, Kingston General Hospital, Stuart Street, Kingston, Ontario, Canada, K7L 2V7

## Abstract

**Introduction:**

Recombinant human activated protein C (APC) therapy has been shown to reduce short-term mortality in patients with severe sepsis. However, survivors of sepsis may have long-term complications affecting health-related quality of life (HRQoL) and resource utilization. The objective of this study was to evaluate prospectively the effect of APC on long-term HRQoL and resource utilization compared with a nonrandomized control group that received standard care.

**Methods:**

This was an observational cohort study at nine Canadian intensive care units. Patients with severe sepsis who survived to 28 days were recruited. Patients who received APC formed the treatment group and those that did not formed the standard care group. Patients who did not receive APC because of central nervous system bleeding risk were excluded from the standard care group. HRQoL (determined using the 36-item Short Form) and resource use were recorded at 28 days, and 3, 5 and 7 months.

**Results:**

One hundred patients were enrolled (64 in the standard care group and 36 in the APC group), with 70 patients completing all follow-up visits. Over the 6 months of follow up, APC-treated patients exhibited statistically significantly better scores for the physical component score (*P *= 0.04) and trends toward improvements in physical functioning (*P *= 0.12), role physical (*P *= 0.10) and bodily pain (*P *= 0.14) as compared with standard care patients. Shorter hospital length of stay was observed for the APC group (36 days versus 48 days; *P *= 0.05).

**Conclusion:**

These findings challenge earlier assumptions suggesting equivalent HRQoL and resource use in APC-treated and standard care patients who survive severe sepsis.

## Introduction

Each year approximately 750,000 patients in the USA develop sepsis, and at least 215,000 of these cases are fatal [1]. Several studies have documented that sepsis is associated with increased hospital resource utilization and prolonged intensive care unit (ICU) and hospital stay [2-5]. With such considerable effects on associated morbidity and mortality, the economic burden associated with sepsis has recently been estimated at 17 billion dollars each year in the USA alone [1]. As novel, expensive therapies for the treatment of sepsis are introduced into international markets, decision makers will need accurate estimates of long-term outcomes and resource utilization if they are to appreciate the relative merits and limitations of these new therapies.

Morbidity associated with severe sepsis can have effects beyond increases in resource consumption. Patients who survive sepsis often have severely compromised organ function that may result in persistent physical and psychological symptoms (dyspnoea, fatigue and depression), impaired functional status (physical, social and emotional function) and reduced health-related quality of life (HRQoL) [6].

In a large randomized clinical trial, recombinant human activated protein C (APC) therapy was shown to reduce 28-day all-cause mortality in patients with severe sepsis from 30.8% in the control group to 24.7% in the treatment group [7]. Long-term follow-up analyses of this large trial have shown improved survival for APC patients with Acute Physiology and Chronic Health Evaluation II scores greater than 25 persisting at 2.5 years [8]. There have been a number of economic evaluations of APC based on 28-day survival data [9-11]. Each has assumed equivalent long-term quality of life and resource use in surviving patients – an assumption that may or may not be valid. Given that APC administration confers persistent long-term effects on overall survival [12] and its proposed relationship with inflammation, organ dysfunction and symptoms/functional status [13], it could plausibly influence long-term HRQoL. The previous literature has suggested that the degree of organ dysfunction during ICU stay can have an impact on HRQoL scores [14], lending support to the hypothesis that APC may influence HRQoL through its effect on organ dysfunction recovery rates. A prospective evaluation of long-term health outcomes and resource use associated with APC would provide useful information and either support or challenge existing assumptions.

The primary objectives of this research were to assess patient's quality of life at 28 days, and 3, 5 and 7 months after treatment with APC, and to compare these data with those from a similar cohort of patients who did not receive APC (we have labelled this control population 'standard care'). The secondary objective was to assess differences in health resource utilization between the two groups. Additionally, we evaluated whether HRQoL following the sepsis episode returned to levels of age-matched Canadians in the general population at 7 months (6 months follow up after day 28 after admission). These data will allow us to assess better the value of novel therapies such as APC to patients, clinicians and policy makers.

## Materials and methods

This study was conducted at nine ICUs at Canadian community and teaching hospitals (see Acknowledgements, below, for a complete list of sites and investigators) during the period from February 2002 to January 2006 (enrolment period plus follow-up phase).

### Study population

Given that randomization of APC was not possible after publication of the Recombinant Human Activated Protein C Worldwide Evaluation in Severe Sepsis (PROWESS) study, our goal was to develop a prospective cohort of patients who survived an episode of severe sepsis and who were similar in all respects except for the receipt, or not, of APC. All patients in the ICU at participating sites were screened daily for the presence of severe sepsis using criteria similar to those used in the PROWESS study (Additional file 1). We note that this protocol differed slightly in that it required two sepsis-induced organ failures, rather than the one required in the PROWESS study. When patients with sepsis were identified, daily data collection was initiated. Decisions to prescribe APC, or not, were at the discretion of the attending physicians based on locally instituted guidelines and their clinical judgement; the research protocol did not influence the use of APC in any measurable way. Patients treated with APC formed the 'treatment' arm and survivors were approached for enrolment in the study.

All standard care (control group) patients had to meet the 'inclusion criteria' for severe sepsis similar to those established by guidelines for enrolment in the large multicenter APC trial [7], including diagnosis of sepsis with two or more organ failures. However, if a septic patient did not receive APC, then they could become a standard care patient if they survived until day 28, at which time they could be approached for inclusion in the study. We excluded from the standard care group those patients who did not receive APC because of central nervous system bleeding risks (history of severe head trauma that required hospitalization, intracranial surgery, stroke within the previous 3 months, or any history of intracerebral arteriovenous malformation, cerebral aneurysm, or central nervous system mass lesion), because these patients probably have or will have a poor HRQoL related to their underlying illness. This exclusion ensured that bleeding risks were similar between APC and standard care groups, because the bleeding risks outlined above are exclusion criteria for APC patients.

### Patient identification

Before ICU discharge or at day 28 from ICU admission (whichever came first), all surviving patients who met the eligibility criteria for the observational study were identified and were approached and asked for their consent to participate in this long-term outcomes study. For patients who were unable to give consent themselves, consent was sought from their surrogate decision maker. Contact information for both the patient and next of kin was obtained. To avoid an imbalance between APC and standard care patients from each site, each participating site enrolled in blocks of three APC and six standard care patients. Sites were not allowed to enrol more than three APC or more than six standard care patients until both blocks were full.

### Study procedures

Upon enrolment, the research coordinator at each site gave the enrolled patient (or surrogate) a package containing study materials. Patients received copies of all data collection tools used including the 36-item Short Form (SF-36) and a diary to keep track of health resource utilization. The research nurse at each site then faxed the enrolment information to the Clinical Evaluation Research Unit at the Kingston General Hospital. From the date of ICU admission, at day 28, and at 3, 5 and 7 months from the date of ICU admission, personnel from the Clinical Evaluation Research Unit contacted the patient or surrogate by telephone. If at any point in the study patients or families lost their study materials, then a new package containing study documents was mailed to them. If the patient was still in hospital at any interval, the local research nurse completed data collection forms with the patient. If the patient was too sick or unable to participate in data collection, study personnel contacted the next of kin requesting them to fill out data collection forms. Surrogate assessments of SF-36 have been shown to be reliable, particularly for assessments of physical function [15].

If researchers were unable to contact the patient after four attempts, or if the telephone number listed was not in service, then the next of kin were contacted. If participants could not be traced through next of kin, the 411 Directory Internet site for each province was searched. Persons who could not be traced through either of these methods were categorized as 'could not contact'.

Baseline data and hospital resource data were collected for both APC and standard care patients. Data collection included important baseline demographics (age, sex, Acute Physiology and Chronic Health Evaluation II score, comorbidity index [16], baseline organ failure and admission diagnosis), process of care variables (concomitant medications, procedures and so on) and outcomes (ICU mortality, length of stay, duration of mechanical ventilation and hospital length of stay). The functional comorbidity index was chosen rather than the Charlson because of the latter's focus on long-term survival and its inability to address HRQoL factors. The functional comorbidity index was developed specifically to correlate with long-term function, not survival.

### 36-Item Short Form

The SF-36 is a multipurpose survey of general health status consisting of eight domains and 36 items. These domains and items were selected from a battery of health status instruments used in the Medical Outcomes Study to represent frequently measured and affected aspects of quality of life [17]. All but one of the 36 items are aggregated into eight subscales, which can also be clustered to form two higher order scales: the physical and mental health component scores. Each subscale is scored from 0 to 100 (100 = optimal). A minimally clinically important difference in the SF-36 is a 5-point difference on the physical function domain or a 2-point difference on the physical subscale [18]. The SF-36 is suitable for self-administration or for administration by a trained interviewer in person or by telephone. The SF-36 has been used in a variety of patient populations, and normal values for age, sex and 14 chronic diseases have been published [18,19] for US populations, as well as more limited data for Canadian populations [20]. Compared with other generic health status instruments, the SF-36 has been shown to have better feasibility, internal consistency, content validity and discriminative ability, and to be more responsive to clinical improvement [21,22]. Recently, Heyland and coworkers [6] demonstrated that the SF-36 has good reliability and validity when used to measure HRQoL in survivors of sepsis.

### Resource utilization

To measure health care resource utilization, study patients were given a diary upon discharge from hospital. Between points of subject contact, the diary was used by the patients to track health resource use. The health resource categories included ICU length of stay, inpatient physiotherapy, emergency visits, physician home visit, outpatient tests, family doctor visit, specialist visit, other medical professional visit, personal care, laundry service, housekeeping, meal preparation, transportation, shopping, respite care, adult day care, occupational therapy, physiotherapy, home nursing, speech language professional, social work, dietary assistance, meal delivery and other services. In addition, the patients were asked to document inability to work because of health reasons and any restrictions in every day activities. At the point of follow up, the interviewer phoned study participants and obtained the information recorded in the diaries.

### Sample size consideration

The primary objective was to detect a 10-point difference in the physical functioning domain of the SF-36. In the context of this study, we expected APC, a novel treatment that modulates the underlying inflammatory disease process in sepsis, to affect the physical outcomes more so than the mental, social, or emotional domains. Although a 5-point difference in physical function is clinically important [18,23], a sample size large enough to detect such a difference was not feasible (estimated *n *= 634). Also, given that APC is a new therapeutic agent associated with significant acquisition cost, a larger treatment effect (10-point difference) was sought to justify its use. Secondary outcomes included the remaining domains of the SF-36 (role physical, bodily pain, general health, vitality, social function, role emotional and mental health) and resource utilization measures. A longitudinal as well as across treatment comparison was undertaken in an attempt to detect any differences across or within groups over time.

We initially planned to enroll 70 patients who survived through to day 28 and who received APC, and a cohort of approximately 140 standard care patients. We anticipated an attrition rate of 20%, resulting in a final target sample size based of approximately 56 APC patients and 112 patients in the standard care arm. We prospectively planned an unbalanced recruitment ratio (1:2), given the anticipated smaller number of APC patients as compared with potential standard care patients.

A previous report showed that the physical functioning domain had a standard deviation of 20.0 points [18]. With the same magnitude of variation anticipated in our study, we anticipated that this sample size would have sufficient power (>86%) to detect a 10-point difference in the physical functioning domain of the SF-36 at any visit, at an α level of 0.05 and based on a two-tailed *t*-test. As a consequence of slow enrolment and insufficient funding, we terminated enrolment after the first 100 patients.

### Statistical methods

Each domain and the two summary component scales of the SF-36 were modelled separately by a linear mixed effects model for repeated measures. This model was estimated by restricted maximum likelihood as implemented in PROC MIXED of SAS (SAS Institute Inc., Cary, NC, USA) [24] and used an unstructured covariance to account appropriately for the repeated measures per subject. Age was added as a continuous covariate to adjust for age differences between treatment groups. The primary analysis used all available observations and treated HRQoL after death as missing. This mixed model does not make the unrealistic assumption that the missing data are similar to the nonmissing data, but rather the less strict assumption that the missing values are similar to nonmissing values for patients of the same age, treatment and observed (pre-death) HRQoL. Thus, the model estimates the expected HRQoL values for the average sample age and the same HRQoL before death or loss to follow up. The robustness of the results to this missing data assumption were assessed by two sensitivity analyses representing two extreme ways of handling deaths: setting all values after death to 0 and including only patients who survived the entire duration of follow up.

Significance values for the baseline demographics and other outcomes were obtained using a Kruskal-Wallis test for continuous variables, the log-rank test for time to return to work, Fisher's exact test for categorical variables with only two levels, and the χ^2 ^test for categorical variables with more than two levels. We considered *P *values of 0.05 and below to be significant, and values below 0.15 to be trending towards significance. All analyses were performed using SAS 9.1 (SAS Institute Inc.).

### Ethics

Consent was obtained from participating patients and their care givers before entry into the study. We obtained research ethics board approval at each participating site.

## Results

### Study sample

**Figure 1:**
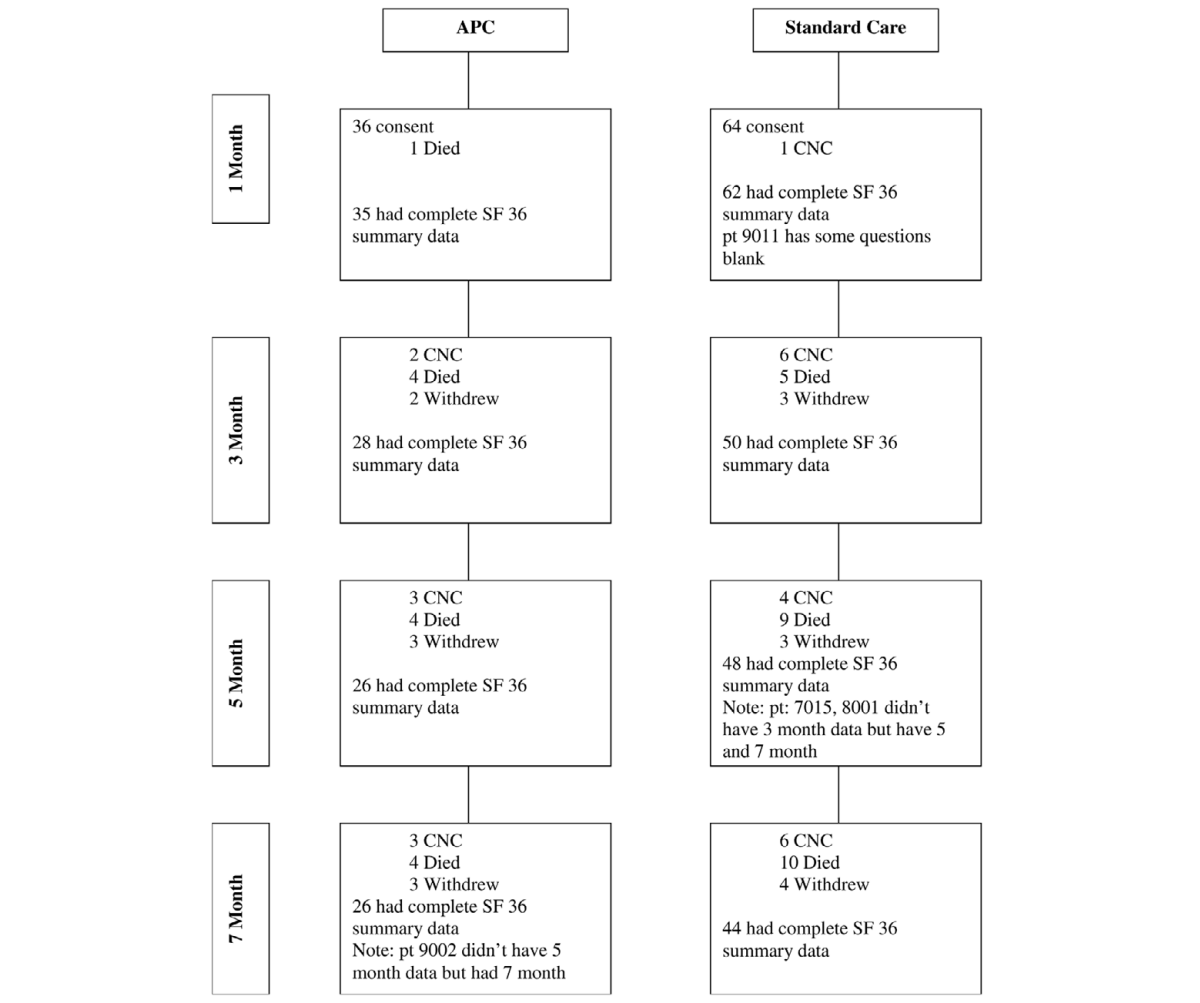
Patient flow diagram for study participants. APC, activated protein C; CNC, could not contact; SF-36, 36-item Short Form.

A total of 164 patients were screened and satisfied the inclusion criteria during the recruitment period; of these, 100 patients gave consent to participate in the follow-up study (36 receiving APC and 64 standard care patients). Among these patients, one died and one could not be contacted during the first month after treatment (Figure 1). These two patients were excluded from all analysis, because no quality of life data were captured past baseline. Baseline characteristics of the two treatment groups for all patients are shown in Table 1. Patients in treatment group (APC) were significantly younger (mean age 54.7 years versus 62.6 years; *P *= 0.03). It was also noted that the APC group tended to have had a greater proportion of admissions from the emergency room (40% versus 27%), and a lower proportion from the ward (20% versus 33%). Baseline severity of illness, comorbidity indices and all other patient characteristics were statistically similar. During the 6-month follow-up period, patients in the treatment group (APC) exhibited a trend toward a lower mortality rate compared with those in the nontreatment (standard care) group (5.7% versus 17.5%; *P *= 0.13), although the difference was not statistically significant, and because of limited sample size we did not control for age differences.

**Table 1:** Demographics for all patients

Parameter	APC (*n *= 35)	Standard care (*n *= 63)	Total (*n *= 98)	*P *values
Age (years; mean ± SD)	54.7 ± 18.1	62.6 ± 15.3	59.8 ± 16.7	0.03
Sex				0.40
Female	19 (54.3%)	28 (44.4%)	47 (48.0%)	
Male	16 (45.7%)	35 (55.6%)	51 (52.0%)	
Admission type (*n *[%])				0.92
Medical	3 (8.6%)	7 (11.1%)	10 (10.2%)	
Surgical	19 (54.3%)	34 (54.0%)	53 (54.1%)	
Missing	13 (38.0%)	22 (35.0%)	35 (36.0%)	
ICU admission diagnosis (*n *[%])				0.30
Cardiovascular/vascular	0 (0.0%)	3 (4.8%)	3 (3.1%)	
Respiratory	7 (20.0%)	18 (28.6%)	25 (25.5%)	
Gastrointestinal	7 (20.0%)	8 (12.7%)	15 (15.3%)	
Neurological	2 (5.7%)	2 (3.2%)	4 (4.1%)	
Sepsis	16 (45.7%)	16 (25.4%)	32 (32.7%)	
Trauma	0 (0.0%)	4 (6.3%)	4 (4.1%)	
Metabolic	0 (0.0%)	2 (3.2%)	2 (2.0%)	
Vascular/cardiovascular	1 (2.9%)	2 (3.2%)	3 (3.1%)	
Orthopaedic	1 (2.9%)	1 (1.6%)	2 (2.0%)	
Missing	1 (3.0%)	7 (12.0%)	8 (9.0%)	
Source of admission (*n *[%])				0.08
ER	14 (40.0%)	17 (27.0%)	31 (31.6%)	
OR elective	0 (0.0%)	5 (7.9%)	5 (5.1%)	
OR emergency	7 (20.0%)	5 (7.9%)	12 (12.2%)	
Other hospital	4 (11.4%)	10 (15.9%)	14 (14.3%)	
Ward	7 (20.0%)	21 (33.3%)	28 (28.6%)	
Missing	3 (8.6%)	5 (7.9%)	8 (8.2%)	
Comorbidites (*n *[%])				0.41
None	29 (82.9%)	51 (81.0%)	80 (81.6%)	
One	6 (17.1%)	9 (14.3%)	15 (15.3%)	
Two	0 (0.0%)	3 (4.8%)	3 (3.1%)	
Functional comorbidity index (mean ± SD)	2.7 ± 2.9	2.1 ± 1.9	2.3 ± 2.3	0.74
APACHE II score (mean ± SD)	27.2 ± 7.5	26.3 ± 7.8	26.6 ± 7.7	0.48

APACHE II, Acute Physiology and Chronic Health Evaluation II; APC, activated protein C; ER, emergency room; ICU, intensive care unit; OR, operating room; SD, standard deviation.

### Heath-related quality of life

**Figure 2:**
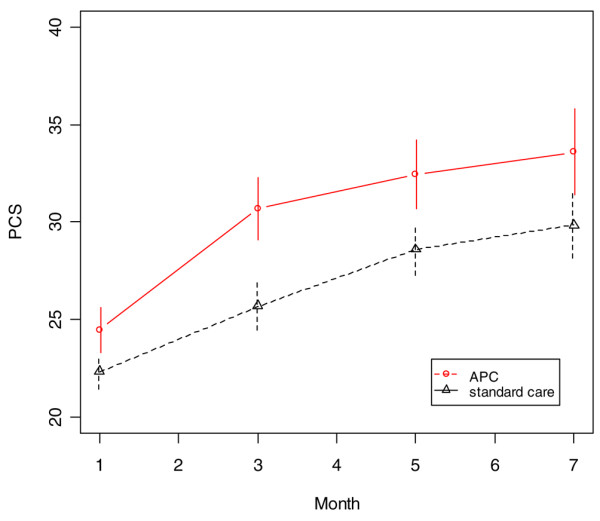
SF-36 physical component scores during the follow-up period (age-adjusted). APC, activated protein C; PCS, physical component score; SF-36, 36-item Short Form.

**Figure 3:**
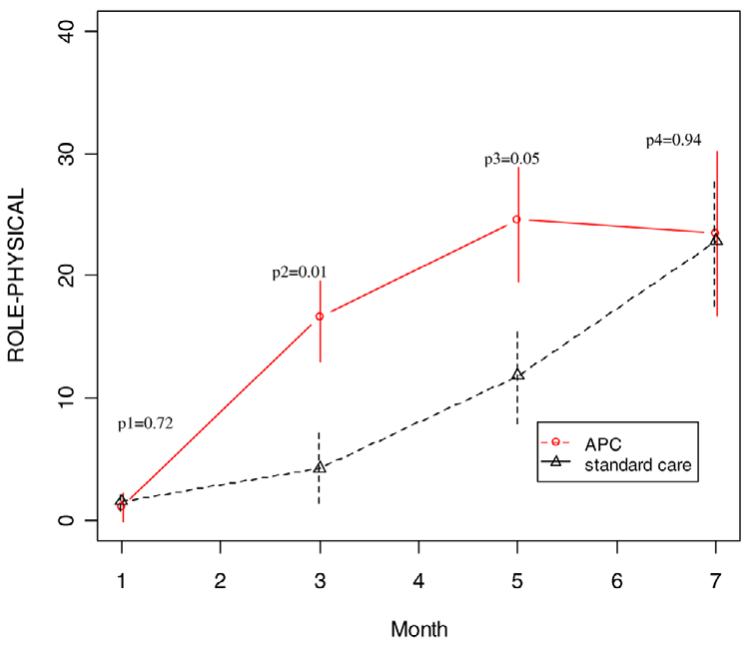
SF-36 role physical scores during the follow-up period (age-adjusted). APC, activated protein C; SF-36, 36-item Short Form.

**Figure 4:**
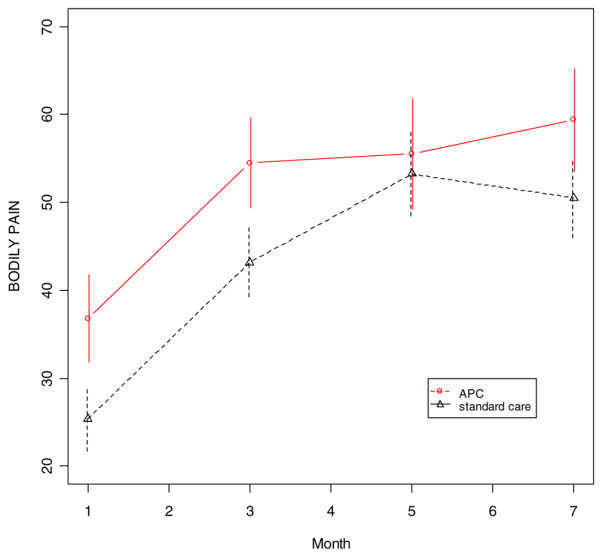
SF-36 bodily pain scores during the follow-up period (age-adjusted). APC, activated protein C; SF-36, 36-item Short Form.

**Figure 5:**
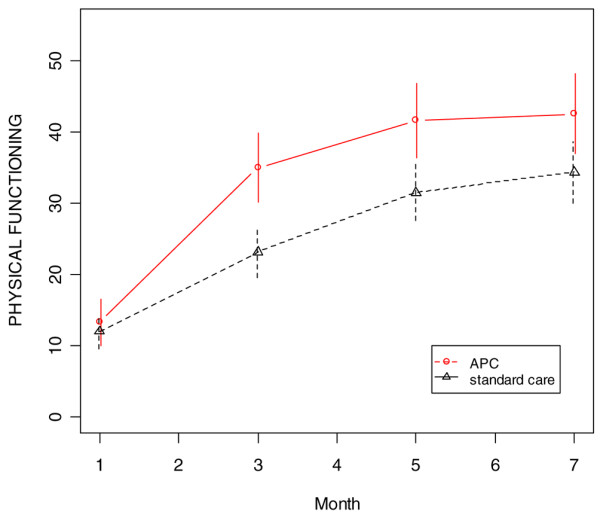
SF-36 physical function scores during follow-up period (age-adjusted). APC, activated protein C; SF-36, 36-item Short Form.

Averaged over the four follow-up assessments and adjusting for age, patients in the APC-treated group had persistently higher physical component score (*P *= 0.04) than did standard care patients (Figure 2). In addition, the APC-treated group had significantly higher role physical scores at months 3 and 5 (*P *= 0.01 and *P *= 0.05, respectively), although the difference averaged over all four assessments did not reach statistical significance (*P *= 0.10) because the groups were nearly identical at months 1 and 7 (Figure 3). There were also trends favouring the APC group for the bodily pain (*P *= 0.14) and the physical function (*P *= 0.12) domains (Figures 4 and 5). The mental component scores and all other domains were similar in the two groups (data not shown).

A sensitivity analysis setting all values after death to 0 provided the following *P *values: *P *= 0.02 for physical component score, *P *= 0.57 for mental component score, *P *= 0.09 for physical function, *P *= 0.05 for role physical, *P *= 0.07 for bodily pain, *P *= 0.24 for general health, *P *= 0.38 for vitality, *P *= 0.36 for social functioning, *P *= 0.86 for role emotional and *P *= 0.54 for mental health. A second sensitivity analysis excluding all patients who did not survive the entire 7 months (which would exclude those lost to follow up) resulted in the following *P *values: *P *= 0.04 for physical component score, *P *= 0.77 for mental component score, for *P *= 0.12 physical function, *P *= 0.20 for role physical, *P *= 0.14 for bodily pain, *P *= 0.38 for general health, *P *= 0.66 for vitality, *P *= 0.52 for social functioning, *P *= 0.96 for role emotional and *P *= 0.93 for mental health. As with our primary analysis, both sensitively analyses confirmed that physical component score was significantly better in the APC group, whereas physical function, role physical and bodily pain exhibited a trend toward statistical significance and none of the other domains suggested any difference between groups.

**Figure 6:**
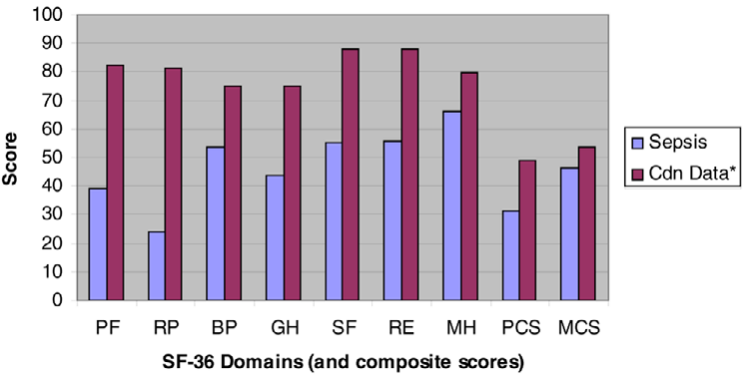
SF-36 scores for sepsis patients 7 months after admission versus Canadian age-adjusted norms. BP, bodily pain; Cdn, Canadian; GH, General Health; MCS, mental component score; MH, mental health; PCS, physical component score; PF, physical function; RE, role emotional; RP, role physical; SF, social functioning; SF-36, 36-item Short Form.

Although patients in both the treatment (APC) and standard care arm of this study showed significant improvement in all domains from baseline (all *P *< 0.01) except general health, at 7 months after the episode of severe sepsis HRQoL was still below that of age-matched controls for Canadians [19] (Figure 6).

### Resource utilization and return to work

Resource utilization was examined for all patients. Patients on treatment (APC) had a shorter length of stay in hospital (36.0 days [interquartile range 21.0 to 58.0 days] versus 48.0 days [interquartile range 31.0 to 78.4 days]; *P *= 0.05). There were no other significant differences for any of the other health services (Table 2).

**Table 2:** Outcomes for all patients

Outcome	APC (*n *= 35)	Standard care (*n *= 63)	Total (*n *= 98)	*P *values
ICU LOS (median [IQR])	14.7 (10.0 to 23.3)	15.2 (9.4 to 29.4)	14.9 (9.6 to 25.5)	0.65
Hospital LOS (median [IQR])	36.0 (21.0 to 58.0)	48.0 (31.0 to 78.4)	42.0 (29.0 to 75.0)	0.05
ICU outcomes (*n *[%])				0.71
Transfer to another acute care facility	1 (2.9%)	3 (4.8%)	4 (4.1%)	
Ward	34 (97.1%)	58 (92.1%)	92 (93.9%)	
Home	0 (0.0%)	1 (1.6%)	1 (1.0%)	
Expired	0 (0.0%)	1 (1.6%)	1 (1.0%)	
Hospital outcomes (*n *[%])				0.91
Transfer to another acute care facility	5 (14.3%)	9 (14.3%)	14 (14.3%)	
Transfer to a chronic care facility	4 (11.4%)	8 (12.7%)	12 (12.2%)	
Home	22 (62.9%)	33 (52.4%)	55 (56.1%)	
Expired	2 (5.7%)	6 (9.5%)	8 (8.2%)	
Missing	2 (5.7%)	7 (11.1%)	9 (9.2%)	
Mortality status at 7 months: dead (*n *[%])	2 (5.7%)	11 (17.5%)	13 (13.3%)	0.13

APC, activated protein C; ICU, intensive care unit; IQR, interquartile range; LOS, length of stay.

Of enrolled patients, 21% were employed before their ICU illness. There was no difference in the number of patients who returned to work overall. Comparing the employment status of the two groups, only a small amount of them (21.4%) were employed before hospitalization. No difference was detected between the two groups in employment status at each time point.

The time to return to employment was compared between groups for the 21 patients who were employed before hospitalization. After 1 month, nine out of 10 (90%) patients in the APC group returned to work, as compared with seven out of 11 (64%) patients in the standard care group. By 7 months all of the patients in the APC group returned to work, as compared with eight out of 11 (73%) patients in the standard care group. Although this is a small sample size, there was a trend suggesting that the APC group returned to work faster (*P *= 0.096).

## Discussion

HRQoL may be an important indicator of overall recovery from a serious illness. This is the first report to compare long-term HRQoL between patients treated with APC and those receiving standard care. Our results suggest that patients treated with APC have improved health outcomes and appear physically to recover more quickly, as compared with those who do not receive APC treatment. Our results apear to be robust under a variety of analysis assumptions.

These findings appear plausible because it is likely that a drug focused on improving the pathophysiology of sepsis will have a continuum of effect on patients, potentially anchored at one end by improvements at a cellular level, but presumably then acting to improve individual organ or multiorgan function, and hopefully leading to a reduction in death rate. It is similarly likely, even in the absence of a survival benefit between groups treated with APC or not, that via beneficial actions at the cellular or organ level APC might lead to more subtle improvements in organ function and recovery that could translate into positive effects on HRQL among those patients who did survive, even without a difference in rate of survival.

These data may challenge the underlying assumption that HRQoL is similar for all survivors in the long term, regardless of treatment regimen. We note that most of the reported economic models employ similar utility scores for APC treated and standard care survivors. Yet other literature suggests a strong correlation between SF-36 scores and utility scores in general populations [25], diabetes [26] and cardiovascular disease [27]. If this holds true in a septic population, then existing literature should be viewed as a conservative estimate of quality-adjusted survival benefit for patients treated with APC. Increased utility scores and reduced resource use may translate into a more favourable incremental cost-effectiveness ratio than has been reported [11-13]. This study has also demonstrated that patients with severe sepsis improve significantly in the 7 months after admission. However, patients surviving severe sepsis, similar to those surviving acute respiratory distress syndrome [28], do not attain equivalent measures of health as compared with age-matched Canadian control individuals [20].

Weycker and coworkers [29] demonstrated that hospitalizations account for the majority of the total medical care costs. Our data on resource use suggest that, at least for the initial hospitalization, patients treated with APC stayed in hospital for shorter periods of time and therefore were less likely to require hospital resources. Although short-term resource use data have been published [30] and demonstrated that older patients treated with APC have statistically significant reductions in ICU days, hospital days, ventilator-free days, and vasopressor-free days, this is the first paper to report on longer term resource use in patients with severe sepsis treated with APC. Although our study did not capture costing data on each of these services, it would be expected that the additional cost of APC would be partially offset by reductions in hospital length of stay.

Our study has limitations that should be noted. Although the intent was to recruit 210 patients, because of limited resources and difficulty in enrolling patients, we were able to enrol only 100 patients during the planned recruitment period. Despite this, there was a statistically significant difference for the physical composite score of the SF-36. A larger sample size might have resulted in a statistically significant finding on those domains exhibiting a trend toward significance. A more comprehensive examination of baseline values, including time to first organ failure, bleeding risk and organ dysfunction, might have allowed us to assess better whether notable differences between groups existed. Hence, the absence of these measures is a possible source of variability between groups. As with any nonrandomized, observational study, bias is possible so that differences between groups may be due to factors other than the presence or absence of treatment. To understand such biases, we captured important baseline demographics (such as age, comorbid illness and underlying severity of illness, among others). The groups appeared to be similar for all measured demographics and disease characteristics except age and source of admission. Age was controlled for in the analysis, but no adjustment was made for source of admission. Although there may be an influence related to source of admission, there is limited literature on its impact on HRQoL. Iapichino and colleagues [31] has shown that ward admissions do increase the chances of mortality (but no effect from emergency admissions), which may correlate with HRQoL scores, but otherwise the impact that source of admission has on HRQoL is unknown. Additionally, we were only able to enrol a portion of all eligible patients (61%). The demographic details on those patients who were screened but elected not to participate were not captured. Also, because there were only a limited number of APC patients available, it is possible that some selection bias could have occurred. To minimize bias sites were instructed to take all consecutive treated patients up to the ratio of 3:6. If they filled there quota of consecutive controls without having enough treated, then they passed on further enrolments to the control group. Enrolment was therefore consecutive with the exception of the control group, so if a selection bias between groups exists it is probably minimal. We recognize that the aforementioned issues related to source of admission and selection bias may create differences between the two groups even after adjusting for age. Because it is possible that these differences could account for some of the observed differences in HRQoL seen between groups, these results should be viewed as suggestive only, and therefore they should be interpreted with caution.

In the case of patients who we were unable to contact at follow-up visits, we employed a standard procedure to contact them. We did not use vital statistics for those patients who we were unsuccessful in contacting. In this regard, we might have overstated the survival statistics, although this would be true for both arms of the study. Also, the fact that an analysis excluding those lost to follow up provided similar results suggested that the differences in loss to follow up between groups had a limited impact on the observed results.

Finally, the generalizability of our observational study reflects that of the patients with severe sepsis at high risk for death, as defined in our protocol. Many patients with severe sepsis may fall outside the eligibility criteria used in the present study, and hence the changes in HRQoL or resource use may be different from our results in the severe sepsis populations not included in this research.

Because this is a small cohort design it may be presumptive in assuming that these populations are identical even after adjusting for age differences. Conversely, it is also inappropriate to assume that the differences in outcomes are purely the result of population differences. A more neutral position, and one that these authors have taken, is that these results are suggestive and warrant further research that will either support or refute these preliminary findings.

## Conclusion

Overall quality of life among patients with severe sepsis showed good recovery in the 7 months after disease diagnosis, but patients did not attain full health, which is consistent with follow up of other forms of critical illness [6]. Patients treated with APC overall appeared to do at least as well or better in HRQoL measurements than those not treated (standard care), and had lower resource use for hospital services. The inferences drawn from these observations admittedly could be challenged given the limitations of a nonrandomized, observational study, and they suggest that further prospective evaluations are warranted. The significance of the findings of this study is that they challenge previous assumptions of equivalence between APC-treated and standard care patients regarding postsepsis quality of life and utility scores. If our results prove to be valid, then the incremental cost-effectiveness ratio of APC may be more favourable than reported in recent publications [11-13]. These findings provide preliminary results that may aid clinicians and decision makers in determining the value of APC and will also be helpful in designing evaluations of future therapies in the management of sepsis. Future phase III follow-up studies should measure HRQoL and utilities for at least up to 6 months after randomization, ideally incorporating the use of propensity scores to improve the matching between the two groups.

## Key messages

• Recombinant human APC effects on long-term HRQoL and resource utilization compared with standard care are largely unknown.

• Study results from 100 matched nonrandomized patients (64 standard care and 36 APC) using the SF-36 as a measure of HRQoL showed that, compared with standard care, patients treated with APC had statistically significantly better physical component Scores (*P *= 0.04) and trends toward statistically significantly better outcomes for physical functioning (*P *= 0.12), role physical (*P *= 0.10) and bodily pain (*P *= 0.14) over a 7-month follow-up period (after admission).

• Shorter length of hospital stay for APC patients compared with standard care was found during this same observation period (36 days versus 48 days; *P *= 0.05).

• Study results for both HRQoL and hospital length of stay highlight that assumptions of similar outcomes (for quality-adjusted life years and resource utilization) for sepsis survivors in cost-effectiveness analyses may be a conservative estimate of APC's benefit over standard care.

• The small sample size and observational nature of this study limit the inferences of this study and further research is warranted to validate the findings.

## Abbreviations

APC = activated protein C; HRQoL = health-related quality of life; ICU = intensive care unit; PCS = physical component score; PROWESS = Recombinant Human Activated Protein C Worldwide Evaluation in Severe Sepsis (PROWESS); SF-36 = 36-item Short Form.

## Competing interests

CJL is a past employee of Eli Lilly Canada Inc. DKH has received both consultancies and grants related to research from Eli Lilly Canada Inc. HNF is currently employed by Eli Lilly Canada Inc. CM has received both honorarium and research grants for research from Eli Lilly Canada. RSF and AGD declare that they have no competing interests.

## Authors' contributions

CJL co-developed the study protocol, assisted in co-ordination of study, undertook background research for manuscript, wrote drafts and obtained author feedback. DKH conceived the study, co-developed the study protocol, undertook primary co-ordination of the study, and participated in writing and revising of the manuscript. HNF assisted in co-ordination of the study, and participated in writing and revising of the manuscript. RSF participated in patient enrolment, and participated in writing and revising of the manuscript. CMM participated in patient enrolment, and participated in writing and revising of the manuscript. AGD provided statistical analysis of results and interpretation, and participated in the writing and revising of the manuscript.

## Supplementary Material

Additional file 1 A Word document outlining the study criteria used to define the presence of severe sepsis. Click here for file
