# Supplementary material for: A long-term follow-up study investigating health-related quality of life and resource use in survivors of severe sepsis: comparison of recombinant human activated protein C with standard care
Source: Crit Care. 2007 Dec 11;11(6):R128. doi: 10.1186/cc6195 (PMC2246225; doi:10.1186/cc6195)
Supplement: Additional file 1 — A Word document outlining the study criteria used to define the presence of severe sepsis. [file cc6195-S1.doc]

APPENDIX A

| **Checklist for Severe Sepsis or Septic Shock** |
| --- |
|  |
| **Infection (1 required; see manual for definitions)** |
| ❑Culture (specify from list) |
| ❑CXR + purulent sputum |
| ❑Clinical Diagnosis (specify from list) |
| **Response (3 required)** |
| ❑T>38 or <36 |
| ❑HR > 90 |
| ❑RR >19 or PaCO2 < 32 or MV |
| ❑WBC > 12 or < 4 or > 10% bands |
| **Organ Failure (2 sepsis-induced required) [see manual for definitions]** |
| ❑SBP < 90 or MAP < 70 or pressors for 1h |
| ❑UO < 0.5 ml/kg/h for 1h |
| ❑PaO2/FiO2 < 250 (<200 if lung is the only dysfunctional organ) |
| ❑Platelets < 80 or 50% decrease |
| ❑pH < 7.30 or lactate > 1.5 upper normal with base deficit > 5 |
| **Bleeding risk** |
| ❑bleeding risk (any of  platelets < 30, elevated INR, elevated PTT) |
| ❑Active postoperative bleeding |
| ❑Severe head trauma |
| ❑Intracranial surgery; stroke within 3 months or history of intracranial AVM |
| ❑Cerebral aneurysm or mass lesion of CNS |
| ❑Congenital bleeding diathesis |
| ❑GI bleeding within previous 6 weeks (unless corrective surgery performed) |
| ❑Trauma considered to increase the risk of bleeding |
| ❑Full dose (therapeutic) anticoagulation |
| ❑Patient received therapeutic anticoagulation in previous 48h |

CXR =Chest X-ray

HR= Heart Rate

RR = Respiration Rate

WBC = White blood cells

SBP= Systolic Blood Pressure

UO= Urine Output

AVM= Arteriole-venous malformation
